# Emergence of *Rickettsia africae*, Oceania

**DOI:** 10.3201/eid1701.101081

**Published:** 2011-01

**Authors:** Carole Eldin, Oleg Mediannikov, Bernard Davoust, Olivier Cabre, Nicolas Barré, Didier Raoult, Philippe Parola

**Affiliations:** Author affiliations: Université de la Méditerranée, Marseille, France (C. Eldin, O. Mediannikov, B. Davoust, D. Raoult, P. Parola);; French Army Health Service, Marseille (B. Davoust, O. Cabre);; Institut Agronomique néo-Calédonien, Païta, New Caledonia (N. Barré)

**Keywords:** Bacteria, vector-borne infections, Oceania, ticks, *Rickettsia africae*, *Amblyomma*, African tick-bite fever, New Caledonia, dispatch

## Abstract

We detected *Rickettsia africae*, the agent of African tick-bite fever (ATBF), by amplification of fragments of *gltA*, *ompA*, and *ompB* genes from 3 specimens of *Amblyomma loculosum* ticks collected from humans and birds in New Caledonia. Clinicians who treat persons in this region should be on alert for ATBF.

Spotted fever group (SFG) rickettsioses are caused by obligate intracellular gram-negative bacteria of the genus *Rickettsia* and are transmitted by hematophagous arthropods, mainly ticks. These zoonoses are important emerging vector-borne infections of humans worldwide. They share characteristic clinical features, including fever, rash, and sometimes an inoculation eschar at the bite site, depending on the rickettsial agent that is involved (*1*).

In Oceania, tick-borne rickettsioses have been reported primarily in Australia. They include Queensland tick typhus (*R. australis*) along the east coast of Australia (*2*), Flinders Island spotted fever (*R. honei*) in southeast Australia (*2*), and variant Flinders Island spotted fever (*R. honei* strain “marmionii”) in eastern Australia (*2*). Furthermore, the DNA of at least 8 incompletely described SFG rickettsiae have been detected in ticks, and the pathogenicity of these rickettsiae remains unknown (*2*). Additionally, *R*. *felis*, the agent of flea-borne SFG rickettsiosis, has been found in Western Australia (*3*), New Zealand (*4*), and recently in New Caledonia (*5*). However, little is known about rickettsioses in the rest of Oceania.

## The Study

From February 2001 to November 2007, tick samples were obtained as part of other, ongoing studies in Oceania. A total of 92 ticks were collected: 14 *Amblyomma loculosum* (13 nymphs, 1 female), including 2 from humans on Chesterfield Island, New Caledonia, and 12 from birds on Walpole Island, New Caledonia; 9 female *A. breviscutatum* from swine on Santo Island, Vanuatu; 2 female *A. laticaudae* from snakes on Pindai Island, New Caledonia; 60 female *Haemaphysalis longicornis*, including 38 from cattle on Santo Island, Vanuatu, 10 from cattle in Port-Laguerre, New Caledonia, and 12 from horses in New Caledonia; and 7 female *Rhipicephalus sanguineus* from dogs in Païta, New Caledonia. Tick species were identified by using taxonomic keys and stored in 70% ethanol before being tested. DNA was extracted from each tick with the QIAamp DNA Mini Kit (QIAGEN, Hilden, Germany) following the manufacturer’s instructions and stored at 4°C until it was used in PCR amplifications. Each sample was tested by quantitative PCR (qPCR) in a LightCycler instrument (Roche Diagnostics GmbH, Mannheim, Germany) for the presence of *Rickettsia* spp. DNA by using primers and Taqman probes (Eurogentec, Seraing, Belgium) that targeted a partial sequence of the citrate synthase (*gltA*) gene (*6*). Tick DNA samples positive by using qPCR were also subjected to standard PCR using CS2d and CS.1258n, which amplify an 1,178-bp fragment of *Rickettsia gltA*. An additional PCR amplification was performed by using primers 190.70, 190.180, and 190.701, which amplify a 629–632-bp fragment of rickettsial *ompA*. Positive controls (*R. montanensis* DNA) and negative controls (sterile water and DNA extracted from uninfected ticks from laboratory colonies) were included in each test. We also amplified and sequenced the 2,113–4,346-bp portion of rickettsial *ompB* (*7*). All amplified products were purified, directly sequenced, and sequences were compared with sequences in the GenBank database.

Positive and negative controls gave expected results in all tests. qPCR and subsequent standard PCR for *ompA* and *gltA* were positive for 3 specimens of *A. loculosum*: 2 nymphs collected from humans on Chesterfield Island, New Caledonia, and 1 female collected from birds on Walpole Island, New Caledonia. The 590-bp amplicons of *ompA* and 1,135-bp amplicons of *gltA* obtained from all samples showed 100% identity with the relevant genes of strain ESF-5 of *R. africae* (GenBank accession no. CP001612.1). A 2,153-bp amplicon of a portion of *ompB* showed 99.8% identity with *R. africae* (strain ESF-5).

Multispacer typing of the detected rickettsiae was performed by using the *dksA-xerC*, *mppA-purC,* and *rpmE-tRNA-fMet* intergenic spacers (*8*). The sequence of the *dksA-xerC* intergenic spacer was found to be 242 bp instead of the 177 bp described for the ESF-5 strain of *R. africae* (GenBank accession no. DQ008280), due to the 65-bp repeat of portion 1–65. A single-nucleotide polymorphism was also found. The *rpmE-tRNA-fMet* (GenBank accession no. DQ008246) spacer showed 100% identity with the *rpmE-tRNA-fMet* spacer of *R. africae*. The *mppA-purC* amplification attempt was negative. Whether this intergenic spacer has been lost or substantially modified remains unknown. Finally, qPCR with primers 1267F/1267R specific for a sequence of the *R. africae* plasmid (*pRA*) produced positive results in 3 ticks infected by *R. africae* (*9*).

## Conclusions

By using molecular criteria (*10*), we identified *R. africae* in an unexpected place and in unexpected tick species. Indeed, *R. africae* is the typical agent of African tick-bite fever. This SFG rickettsia is prevalent mostly in sub-Saharan Africa, where vectors are ticks of the genus *Amblyomma* (mainly *A. hebraeum* and *A. variegatum*) (*11*). The rates of *R. africae* infection in ticks in ATBF-endemic areas are typically high and may reach 100% (*11*). These ticks have also been shown to act as reservoirs for *R. africae* (*12*). ATBF occurs 5 to 10 days after the bite of a tick infected with *R. africae* (*1*). Clinical features include an inoculation eschar, frequently more than one, predominant on the lower limbs; fever; regional lymphadenopathy; and a rash that is sometimes vesicular (*1*).

In our study, 2 of 3 amplified and sequenced genes showed 100% identity with *R. africae*, and a third gene (*ompB*) showed a degree of difference less than that needed to determine a new species (*10*). Thus, our findings demonstrate the existence of a variant *R. africae,* despite the previously identified clonality of its strains in Africa (*9*) and the West Indies, where it was carried by *A. variegatum* ticks from western Africa in the 18th century (*1*).

New Caledonia is a French territory (249,000 inhabitants; 18,575.5 km^2^ of land area) located in the subregion of Melanesia in the southwest Pacific. Chesterfield Island and Walpole Island belong to the archipelago and are uninhabited coral sand cays colonized by marine birds. We detected *R. africae* in *A. loculosum* ticks in Oceania. This tick is known to infest marine birds that are distributed on numerous tropical islands of the southern oceans (*13*). *A. loculosum* ticks have been found in the Indian Ocean (Tanzania, Seychelles Islands, Mauritius, Cocos [Keeling] Island group, Madagascar), on the Coral Sea Islands (Queensland, numerous islands and reefs), near New Caledonia (Surprise Island), and in the Caroline Group in the Pacific Ocean (*13*). Other hosts are goats (Australia) and lizards (Seychelles) (*13*). Aride virus, an ungrouped arbovirus, has been isolated from *A. loculosum* ticks collected from dead birds in the Seychelles, which suggests that this tick may be a vector of bird infections (*14*). *A. loculosum* ticks are also known to readily feed on humans (Seychelles, Australia, New Caledonia, Caroline Islands) (*13*).

Two of the 3 specimens of *A. loculosum* ticks that tested positive for rickettsiae were collected from humans who worked with birds on Chesterfield Island, but those persons only remained on the island for a short period. The duration of tick attachment was not known, but to the best of our knowledge, no illness developed after these tick bites. Further investigations are necessary to evaluate the prevalence and distribution of *R. africae* in New Caledonia and in other islands of Oceania, as well as its interactions with *A. loculosum* ticks. However, clinicians in Oceania and around the world should be aware of the presence of *R. africae* in New Caledonia, an island increasingly visited by tourists from all over the world, and in other islands where *A. loculosum* ticks are prevalent.
